# Bayesian changepoint detection for epidemic models

**DOI:** 10.1038/s41598-025-01944-w

**Published:** 2025-07-01

**Authors:** Peter Johnson, Jesper Lund Pedersen

**Affiliations:** 1 Department of Mathematics, The University of Manchester, Manchester, M13 9PL UK; 2 Department of Mathematical Sciences, University of Copenhagen, Copenhagen, 2100 Denmark

**Keywords:** Viral infection, Epidemiology

## Abstract

This paper demonstrates how Bayesian stochastic filtering techniques can be used to detect changepoints in the transmission rate, as well as identify the rate itself, in the spread of disease using the susceptible-infectious-recovered (SIR) model. To better model real-world scenarios, a stochastic SIR model is considered where the transmission rate is unknown a priori, the number of people moving between compartments is perturbed by additional randomness, and the rate changes at unknown points in time. Changepoints can be used to model disruptions in disease spread, such as those caused by public health measures or new variants. We consider this problem in a Bayesian setting, where the unknown rate and changepoints are modelled as random variables with known prior distributions. This rate can be observed indirectly via the drift of a Brownian motion, before optimally filtering the transmission rate along with any changepoints using Bayesian stochastic filtering techniques. The methods are illustrated with an example using a real dataset from the COVID-19 pandemic, effectively detecting changepoints related to public health measures and the spread of the Omicron variant in the United Kingdom.

## Introduction

In infectious diseases models, the transmission rate is a key epidemiological parameter. In basic models, it is typically assumed to stay constant throughout the outbreak of the disease. However, there are several ways in which the transmission rate may change over time. Two examples are

1. Public health measures: One of the most effective ways to reduce disease transmission is by decreasing the number of contacts an infected individual has through social distancing and national lockdowns, thereby directly reducing the transmission rate.
2. New variants of the disease: These might be more transmissible due to a higher probability of a given contact catching the disease, thus increasing the transmission rate.

In this paper, we propose a Bayesian framework that incorporates such changepoints, where the transmission rate is considered as an unknown parameter having a known prior distribution, and similarly allows for unknown changepoints. Using continuous-time Bayesian stochastic filtering techniques, we develop a method for Bayesian estimation of the transmission rate and detection of changepoints. We demonstrate the applicability of the proposed methodology using a COVID-19 pandemic dataset from the United Kingdom.

The proposed model is based on the deterministic susceptible-infectious-recovered (SIR) model, a popular compartmental model for the spread of disease^1^. The SIR model is a special case of the Kermack-McKendrick model^2^ and has been applied to model diseases like Ebola^3^, vaccination strategies^4^, and even the spread of information^5^ or the popularity of a song^6^.

Real epidemic data tends to be much noisier than the processes observed in this deterministic model, particularly at relatively low levels of infection. To reflect this, random variation is often introduced to the compartmental model^7^. Numerous papers have aimed to estimate unknown transmission rates in both deterministic^8,9^ and stochastic^10^ models. One approach to introduce stochasticity into the SIR model is the addition of Brownian noise to the deterministic model^11–14^ which enables the use of techniques from stochastic calculus and developments related to other areas, including stochastic filtering analysis. This approach is detailed in Section “Stochastic SIR model with changepoints”.

The modelling should also be sufficiently flexible enough to respond to changing human behaviour or variation in the pathogen^15,16^. To achieve this, we incorporate changepoints combined with Bayesian unknown parameters in the transmission rate, making it time-dependent. A crucial observation in this paper is that the observed process in the Bayesian stochastic SIR model can be transformed into a simple Brownian motion with drift, where the time dependent transmission rate appears as the drift term.

Another approach to introduce stochasticity and include changepoints in compartmental models is to consider a discrete-time approximation of the SIR model^17,18^, where transitioning between states is governed by probabilities. Markov chain Monte Carlo (MCMC) is then used to perform Bayesian inference on changepoints.

The proposed method incorporating changepoints addresses the inferential challenges of the Bayesian stochastic SIR model and is based on changepoint analysis originating in ^19^. A vast literature exists on different methods for changepoint detection, and a review of various methods can be found in ^20^. There are two essential elements for a changepoint detection method: a model to fit between changepoints and a strategy for identifying the changepoints. For models with a single changepoint, where the model is known both before and after the unknown changepoint, existing methods minimize the delay while maintaining a given level of accuracy. These methods are well-studied^21^ and known as quickest detection problems in the field of optimal stopping problems.

For many applications where the model between changepoints is unknown, MCMC methods are commonly used to detect changepoints in sequential Bayesian models^22^. Numerous previous studies on changepoint detection in epidemic models^17,18^ have also applied MCMC methods within this framework. Additional methods for use with changepoint analysis include iterative sequential regression, generalised additive models, maximum likelihood estimation, and Kruskal-Wallis testing^23,24^.

In the proposed continuous-time Bayesian stochastic SIR model, the transmission rate is considered to be the signal process that cannot be observed directly. Bayesian stochastic filtering techniques (cf. ^25^, Section 3.3) can then be applied to estimate the unknown signal, specifically to estimate transmission rates and detect any changepoints if present. This method has the benefit of providing full inference of the model’s unknown parameters via the posterior joint distribution, and can be used sequentially to quantify key values in real-time, such as the posterior probability that the observed data contains a given number of changepoints.

The exposition of the material is organised as follows. In Section “Stochastic SIR model with changepoints”, we introduce the (deterministic) SIR model and propose a Bayesian stochastic SIR model with changepoints in the transmission rate (and the recovery rate). In Section “Bayesian stochastic filtering”, we discuss how, using continuous-time Bayesian stochastic filtering techniques, we can define the posterior joint distribution, which is the updated distribution given the observed data for the unknown rates and changepoints. These distributions are then used to provide the posterior distribution of the reproduction rates and the maximum a posteriori probability (MAP) estimates for the unknown parameters. Section  “Simulation study” details the performance of the methodological proposals, evaluated through a simulation study. In Section “Application to COVID-19 data”, we apply these methods to data from the COVID-19 pandemic in the UK, detecting changes related to public health measures and the emergence of the more transmissible^26^ Omicron variant.

## Stochastic SIR model with changepoints

In this section, we describe the model used for changepoint detection of the transmission rate in detail. We begin by introducing some notation and assumptions used throughout the paper. We assume that the observations (data) from the spread of the disease are as follows

$$\begin{aligned} S_t&: \text{ number } \text{ of } \text{ people } \text{ susceptible } \text{ to } \text{ the } \text{ disease } \text{ at } \text{ time } t; \\ I_t&: \text{ number } \text{ of } \text{ people } \text{ infected } \text{ with } \text{ the } \text{ disease } \text{ at } \text{ time } t; \\ R_t&: \text{ number } \text{ of } \text{ people } \text{ that } \text{ have } \text{ recovered } \text{ from } \text{ the } \text{ disease } \text{ at } \text{ time } t; \end{aligned}$$

where $N_t = S_t + I_t + R_t$ is the population size at time *t*. The assumptions are that anyone born into the population is susceptible, individuals in the infected group have the disease and are infectious to others, and those who have recovered are no longer susceptible. Throughout the paper, we assume that the population size $N_t$ is known/given, as well as the birth rate $\Lambda$, individuals born into the susceptible group, and the death rate $\mu$ (both rates with respect to units of time, specifically per day).

### Deterministic SIR model

We initially consider a well-established deterministic compartmental model for disease spread. Thus, the susceptible-infectious-recovered (SIR) model is given by the following deterministic dynamics

1 $$\begin{aligned} dS_t&=\Big ( \Lambda N_t -\mu S_t -\beta \frac{ I_t S_t}{N_t} \Big )\,dt \end{aligned}$$

2 $$\begin{aligned} dI_t&= \Big (\beta \frac{I_t S_t}{N_t} -(\gamma + \mu ) I_t \Big )\,dt \end{aligned}$$

3 $$\begin{aligned} dR_t&= \big (\gamma I_t -\mu R_t\big )\,dt \end{aligned}$$

with $N_t = S_t + I_t + R_t$ or equivalently $dN_t = dS_t + dI_t + dR_t$. The model’s parameters are all rates with respect to units of time considered in the following discussion as per day: $\beta$ is the transmission rate, the average number of daily contacts multiplied by the probability of passing the disease, and $\gamma$ is the recovery rate, where a person spends on average $1/\gamma$ days as infected before recovering. It can be noted that the death rate is equal for all compartments and if the birth and death rates are equal ($\Lambda = \mu$), then the population size is constant. For further discussion, see for example ^1^, Chapter 2 and ^27^, Chapter 2.

The transmission rate $\beta$ is an important quantity in the model and can be constructed as the number of contacts an infected individual has (per day) multiplied by the probability of a given contact catching the disease. This means the number of individuals moving from susceptible to infected is $\beta S_tI_t/N_t$, as seen in the dynamics of (1) and (2). This represents the product of: the number of contacts an infected individual has, the probability of a given contact catching the disease, the proportion of the contacts which are susceptible, and the number of such infected individuals.

### Stochastic SIR model with changepoints

As mentioned in the Introduction, we expand the deterministic SIR model in two directions. Firstly, the number of people passing between compartments is perturbed by some noise. Specifically, the SIR dynamics (1)-(3) gain an additional Brownian noise term (cf. ^28^, Equation (4) and ^13^, Page 39). Secondly, we adopt a Bayesian approach to model the transmission rate (and recovery rate) incorporating changepoints. In this formulation, the transmission rate (and recovery rate) is a stochastic process, given in terms of random variables with appropriate prior distributions.

Thus, the stochastic SIR model is given by the following stochastic differential equations

4 $$\begin{aligned} d {S}_t&= \left( \Lambda N_t -\mu {S}_t -\beta _t \frac{I_t {S}_t}{N_t} \right) dt -\sigma _1 \frac{I_t S_t}{N_t}\, d B^{(1)}_t \end{aligned}$$

5 $$\begin{aligned} d {I}_t&= \left( \beta _t \frac{I_t S_t}{N_t} -(\gamma _t + \mu ) I_t \right) dt +\sigma _1\frac{I_t S_t}{N_t}\, d B^{(1)}_t - \sigma _2 I_t\, d B^{(2)}_t \end{aligned}$$

6 $$\begin{aligned} d {R}_t&= \left( \gamma _t I_t -\mu R_t\right) dt + \sigma _2 I_t\, d B^{(2)}_t\ \end{aligned}$$

where $B^{(1)}$ and $B^{(2)}$ are two independent standard Brownian motions with $\sigma _1$ and $\sigma _2$ as given constants. The stochastic transmission rate $\beta _t$ and the stochastic recovery rate $\gamma _t$ are defined as

7 $$\begin{aligned} \beta _t = \sum _{i=1}^n b_{i-1} I_{[\theta _{i-1},\theta _{i})}(t) + b_{n} I_{[\theta _{n},\infty )}(t) \text { and } \gamma _t = \sum _{i=1}^{m} g_{i-1} I_{[\bar{\theta }_{i-1},\bar{\theta }_{i})}(t) + g_{m}I_{[\bar{\theta }_{m},\infty )}(t) \end{aligned}$$

where the parameters (random variables) ${\textbf {b}}=(b_0,b_1,\ldots ,b_n)$ with known joint prior distribution are independent of the changepoints $\varvec{\theta }=(\theta _0,\theta _1,\ldots ,\theta _n )$ satisfying $0=\theta _0<\theta _1<\cdots <\theta _n$ and $\theta _i-\theta _{i-1}$ are independent and identically distributed with a known prior distribution for $i=1,\ldots ,n$. In the same way, the parameters ${\textbf {g}}=(g_0,g_1,\ldots ,g_{m})$ with known joint prior distribution are independent of the changepoints $\varvec{\bar{\theta }}=(\bar{\theta }_0,\bar{\theta }_1,\ldots ,\bar{\theta }_{m} )$ satisfying $0=\bar{\theta }_0<\bar{\theta }_1<\cdots <\bar{\theta }_m$ and $\bar{\theta }_i-\bar{\theta }_{i-1}$ are independent and identically distributed with a known prior distribution for $i=1,\ldots ,m$.

Some further remarks for the Bayesian model described are:

1. The motivation for perturbing the movement of individuals between compartments with some noise is to better represent real data, which does not follow a simple deterministic path that would allow us to perfectly predict future values of (*S*, *I*, *R*).

2. The motivation for making the transmission rate (and recovery rate) a stochastic process (7) is to address substantial research suggesting that modelling has often not been flexible enough to respond to changing human behaviour or variation in the pathogen^15,16^.

3. In practice, when little or nothing is known about the prior distributions of the Bayesian parameters, it is standard in a Bayesian setting to use priors with some characteristics (moments, quantiles, etc.) that maximise entropy over the variable’s range, e.g. Gaussian distribution on ${\mathbb {R}}$, exponential distribution on ${\mathbb {R}}_+$, or uniform distribution on a given interval.

4. In the model, *n* is the maximum number of changepoints which could be identified in the data. However, at a given time of observation *t*, it may be that no changepoints have yet been observed and the Bayesian stochastic filtering described in Section “Bayesian stochastic filtering” can confirm this. Hence the choice of *n* is not prescriptive and the model is robust as long as the number of changepoints in the observed data does not exceed *n*. While choosing a large *n* is preferable, it increases the dimensionality of the unknowns and thus the computational time required for filtering.

5. Although the main focus of the paper is on the transmission rate, the recovery rate in the model is also time-dependent. New variants of the disease or new therapeutic medicines that reduce the infective period are examples where the recovery rate might change over time.

The value of the transmission rate is not directly observable due to the noise in the data. Through observation of the path $(S_u,I_u,R_u;0\le u\le t)$ we wish to infer $({\textbf {b}}, \varvec{\theta })$, via the posterior (joint) distribution, and so inferring the path of the transmission rate $(\beta _u; 0\le u\le t)$. A key observation of the paper is that the observed processes (*S*, *I*, *R*) solving (4)-(6) can be transformed directly to the two observable processes $X^{(1)}$ and $X^{(2)}$ which solve

8 $$\begin{aligned} dX^{(1)}_t&=\frac{N_t}{I_t} \left( \Lambda \frac{ N_t}{ S_t}-\mu \right) dt -\frac{N_t}{I_t S_t}\, d S_t= \beta _t\, dt+\sigma _1\, d B^{(1)}_t \end{aligned}$$

9 $$\begin{aligned} dX^{(2)}_t&= \mu \frac{ R_t}{I_t}\,dt +\frac{1}{ I_t}\,d R_t = \gamma _t\, dt+\sigma _2\, d B^{(2)}_t. \end{aligned}$$

Thus, $X^{(1)}$ and $X^{(2)}$ are two Brownian motions with drift, where the drift is the transmission rate and recovery rate, respectively. If a priori all the random variables defined are mutually independent, along with being independent from $B^{(1)}$ and $B^{(2)}$, then the two processes $X^{(1)}$ and $X^{(2)}$ are independent, and the analysis of the transmission rate can be achieved independently of the analysis for the recovery rate and vice versa. In this setting, it is apparent that observation of $X^{(2)}$ will hold no information for inferring the transmission rate $\beta _t$ and equally observation of $X^{(1)}$ will hold no information for inferring the recovery rate $\gamma _t$. The above framework for the SIR model can be extended by adding further compartments to the model such as the SEIR or SEIRS model^29^ which includes an exposed group where individuals have the disease but are not yet infectious themselves, i.e. there is an incubation period. It is also applicable to more advanced models like the SEEIIR model, which employs multiple exposed and infectious compartments to approximate Erlang-distributed incubation and infectious durations.

As mentioned above, the value of $\beta _t$ and $\gamma _t$ are not directly observable due to the noise and so can only be inferred. While the values of $\sigma _1$ and $\sigma _2$ are given by the following formulas. By applying Itô’s formula on $S_t^2$ and $R_t^2$, we get that $\sigma _1$ and $\sigma _2$ can be expressed in terms of the observed processes (*S*, *I*, *R*) via

10 $$\begin{aligned} \sigma _1^2 = \frac{\displaystyle {S_t^2 - S_0^2 - 2 \int _0^t S_u\, d S_u}}{\displaystyle {\int _0^t I_u^2 S_u^2 N_u^{-2}\, du}} \ \text { and } \ \sigma _2^2 = \frac{\displaystyle {R_t^2 - R_0^2 - 2 \int _0^t R_u\, d R_u}}{\displaystyle {\int _0^t I_u^2\, du }}. \end{aligned}$$

The first expression is used to estimate $\sigma _1$ in the data analysis of the COVID-19 pandemic data from the UK in Section “Application to COVID-19 data”.

## Bayesian stochastic filtering

In this section, we study procedures for estimating the unknown transmission rate, specifically changepoint detection procedures, and estimating the value of the transmission rate between changepoints. We consider the Bayesian stochastic SIR model with changepoints as given in Section “Stochastic SIR model with changepoints”. For simplicity in the following discussion, we will consider the case of a single changepoint ($n=1$) in the transmission rate. In this case, the transmission rate is given by

$$\begin{aligned} \beta _t = b_0 I_{[0,\theta )} (t) + b_1 I_{[\theta ,\infty )}(t) \end{aligned}$$

where $b_0$, $b_1$, and $\theta$ are random variables with prior distributions denoted by

$$\begin{aligned} F_i^{b}(x)= {\mathsf{P}}(b_i \le x)\ &\text { and }\ f_i^{b}(x)\,dx=dF_i^{b}(x)={\mathsf{P}}(b_i\in dx) \text{ for } i= 0,1\\ F^\theta (t) = {\mathsf{P}}(\theta \le t)\ &\text { and }\ f^{\theta }(t)\,dt=dF^{\theta }(t) ={\mathsf{P}}(\theta \in dt). \end{aligned}$$

These random variables, along with the two Brownian motions $B^{(1)}$ and $B^{(2)}$, are assumed to be mutually independent. However, the ideas can be easily extended to higher dimensions, incorporating more changepoints with more transmission rates, or equally applied to estimate the recovery rate from observing $X^{(2)}$. The same approach presented below for estimating and detecting changepoints in the transmission rate can also be applied to estimate and detect changepoints in the recovery rate.

From observing the process $X^{(1)}$ defined in (8), we can apply Bayesian stochastic filtering techniques (cf. ^25^, Section 3.3) to define key quantities and estimates for the underlying random variables. Below is a brief outline of the formulas used to calculate the estimates for the transmission rate(s) and any changepoint if present. In the following, we define the likelihood processes and their integrals as follows

11 $$\begin{aligned} L^{(1)}_{t}(y)&= \exp \left( \frac{y}{\sigma _1} X^{(1)}_t- \frac{1}{2}\frac{y^2}{\sigma _1^2} t \right) \end{aligned}$$

12 $$\begin{aligned} L^{(1)}_{[s,t)}(y)&= \exp \left( \frac{y}{\sigma _1}\big ( X^{(1)}_t-X^{(1)}_s\big ) - \frac{1}{2}\frac{y^2}{\sigma ^2_1} \big (t-s\big ) \right) \end{aligned}$$

13 $$\begin{aligned} {\bar{L}}^{(1,0)}_{t}&= \int _0^\infty L^{(1)}_t(x)\ d F^{b}_0(x)=\int _0^\infty L^{(1)}_t(x)f_0^{b}(x)\,dx \end{aligned}$$

14 $$\begin{aligned} {\bar{L}}^{(1,1)}_{t}&= \int _0^\infty L^{(1)}_t(x)\ d F^{b}_1(x) = \int _0^\infty L^{(1)}_t(x)f_1^{b}(x)\,dx \end{aligned}$$

15 $$\begin{aligned} {\bar{L}}^{(1)}_{[s,t)}&= \int _0^\infty L^{(1)}_{[s,t)}(x)\ d F^{b}_1(x)=\int _0^\infty L^{(1)}_{[s,t)}(x)f_1^{b}(x)\,dx. \end{aligned}$$

Additionally, let the process ${{\tilde{Z}}}_t$ be defined as

$$\begin{aligned} {{\tilde{Z}}}_t =\int _0^\infty \int _0^\infty \int _0^\infty \left( L_t^{(1)}(x_0) I_{[0,s)} (t) +L_s^{(1)}(x_0) L^{(1)}_{[s,t]}(x_1)I_{[s,\infty )}(t)\right) f^b_0(x_0) f^b_{1}(x_1) f^\theta (s)\,dx_0\, dx_1\, ds. \end{aligned}$$

It should be noted here that the likelihood process $L^{(1)}$ is only given in terms of the observed process $X^{(1)}$ as $\sigma _1$ is given by the expression in (10). This means in this setting, the likelihood is observable, which is exploited below. In the general Bayesian filtering framework, these likelihoods (11)-(15) can also include the unobservable process from the drift term (see ^25^, Equation (3.18)), in this case, $\beta _t$, which would be problematic.

### Posterior distributions

Before we start observation, the prior distributions provide the best estimate of where the outcomes of the random variables $(b_0,b_1,\theta )$ are likely to be. However, as we start observing $X^{(1)}$, we are provided with more information about the outcomes $(b_0,b_1,\theta )$, as they will directly affect the drift of this process. Hence, through observation of $X^{(1)}$, we can update the best estimate of where the outcomes of the random variables $(b_0,b_1,\theta )$ are likely to be, giving the posterior joint distribution.

The posterior joint distribution for $(b_0,b_1,\theta )$ given the observed path of $X^{(1)}$ from time 0 to time *t*, denoted $(X^{(1)}_u; 0\le u\le t)$, is defined as

16 $$\begin{aligned} {\mathsf{P}}\big (b_0\in dx_0,b_1\in dx_1,\theta \in ds\,\big |\, (X^{(1)}_u;0\le u\le t)\big )= \hat{p}_t( x_0, x_1,s )\ dx_0\ dx_1\ ds \end{aligned}$$

where ${\hat{p}}_t$ is given as follows

17 $$\begin{aligned} \hat{p}_t(x_0, x_1,s)= \frac{ L^{(1)}_t(x_0)I_{[0,s)}(t) + L^{(1)}_s (x_0) L^{(1)}_{[s,t)}(x_1) I_{[s,\infty )}(t) }{{{\tilde{Z}}}_t} f^b_0(x_0) f^b_{1}(x_1) f^\theta (s). \end{aligned}$$

This is a very important distribution as it can be used to find the most likely set of parameters which fit the data (see Section “Parameter estimates”), and it can also be used to find the marginal posterior distributions of the individual random variables by integrating with respect to the others. All the marginal posterior distributions can be calculated from (16), as well as some other important qualities which we shall discuss below. Starting with the marginal posterior density for the changepoint, denoted ${\hat{f}}^\theta _t$, is given as follows

18 $$\begin{aligned} {\hat{f}}^\theta _t(s)=\frac{ {\mathsf{P}}\big (\theta \in ds\,\big |\, (X^{(1)}_u;0\le u\le t) \big ) }{ds}= \frac{ {\bar{L}}_t^{(1,0)} I_{[0,s)}(t) + {\bar{L}}_s^{(1,0)}{\bar{L}}^{(1)}_{[s,t]}(s) I_{[s,\infty )}(t) }{{{\tilde{Z}}}_t}f^\theta (s). \end{aligned}$$

This also gives the useful quantity of the probability that a changepoint has occurred in the observed data

19 $$\begin{aligned} \Pi _t = {\mathsf{P}}\big ( \theta \le t\,|\, (X^{(1)}_u;0\le u\le t) \big )=\int _0^t {\hat{f}}^\theta _t(s)\, ds. \end{aligned}$$

In a similar way, we can calculate the marginal posterior densities for $b^{(0)}$ and $b^{(1)}$ given everything observed up to time *t* as follows

$$\begin{aligned} {\hat{f}}^b_{0}(t, x_0)&=\frac{{\mathsf{P}}\big (b_0 \in dx_0 \,\big |\, (X^{(1)}_u; 0\le u\le t) \big )}{dx_0}= \frac{\displaystyle { \big (1-F^\theta (t)\big ) L_t^{(1)}(x_0) + \int _0^t L_s^{(1)}(x_0) {\bar{L}}^{(1)}_{[s,t]} f^\theta (s)\,ds}}{{{\tilde{Z}}}_t} f^b_{0}(x_0) \\ {\hat{f}}^b_{1}(t,x_1)&=\frac{{\mathsf{P}}\!\big (b_1 \in dx_1\,\big |\, (X^{(1)}_u;0\le u\le t) \big )}{dx_1}=\frac{\displaystyle {\big (1- F^\theta (t)\big ) {\bar{L}}_t^{(1,0)}(x_1) + \int _0^t {\bar{L}}_s^{(1,0)} L^{(1)}_{[s,t]}(x_1)f^\theta (s)ds}}{{{\tilde{Z}}}_t} f^b_{1}(x_1) \end{aligned}$$

and finally, the posterior density of the current transmission rate at time *t*, $\beta _t$, as

$$\begin{aligned} {\hat{f}}^\beta (t,x) =\frac{{\mathsf{P}}\big (\beta _t \in dx\,\big |\, (X^{(1)}_u; 0\le u\le t)\big )}{dx}= \frac{\displaystyle { f^b_{0}(x) \big (1-F^\theta (t)\big ) L_t^{(1)}(x) +f^b_{1}(x) \int _0^t {\bar{L}}_s^{(1,0)} L^{(1)}_{[s,t]}(x) f^\theta (s)\,ds }}{{{\tilde{Z}}}_t}. \end{aligned}$$

### Parameter estimates

The unknown parameters in the model can be estimated in a number of ways. Two important questions can be asked about the unknown rates (e.g., transmission, recovery, basic reproduction (see (22) below)) at time *t* given the observations up to that time:

1. What is the best estimate of the historical path of the unknown rates?
2. What is the best estimate of the current unknown rates?

Using the posterior distributions from Section “Posterior distributions”, we can find a number of ways to estimate the unknown outcomes of the random variables in the model. The most appropriate estimate depends on which of the above cases is being considered.

In the first case, the most natural estimates to use would be the maximum a posteriori probability (MAP) estimate for $(b_0,b_1,\theta )$. This would be the set of outcomes which maximise the posterior joint density (17), such that the MAP estimate of the unknowns in the model at time *t* is defined as

20 $$\begin{aligned} (b_0^*,b_1^*, \theta ^*)_t = {{\,\mathrm{arg\,max}\,}}_{(x_0,x_1,s)} \hat{p}_t( x_0, x_1, s). \end{aligned}$$

The MAP estimate better accounts for the interconnection between these outcomes and the following path

21 $$\begin{aligned} b^*_0 I_{[0,\theta ^*)} (u) + b^*_1 I_{[\theta ^*,\infty )}(u) \text { for } u \le t \end{aligned}$$

would provide sensible fitting to the observed data and an estimate of the path of $\beta _t$ from time 0 to time *t*, denoted as $(\beta _u;0\le u\le t)$. This would not necessarily be the case if the individual conditional expectations of random variables, calculated using the marginal posterior distributions, were used to fit the data.

In the second case, estimating the current value of the transmission rate $\beta _t$, it is more natural to consider the conditional expectation, which gives the minimum mean square error (MMSE) for the true value given the observed data. If the recovery rate $\gamma$ is assumed to be a known constant, estimating the transmission rate $\beta _t$ is equivalent to estimating the basic reproduction rate given by

22 $$\begin{aligned} R^{0}_t=\frac{\beta _t}{\gamma +\mu }. \end{aligned}$$

The posterior density of $R_t^0$ is

23 $$\begin{aligned} {\hat{f}}^R_0(t,x)=\frac{{\mathsf{P}}\big (R_t^0 \in dx\,\big |\, (X^{(1)}_u; 0\le u\le t)\big )}{dx}=(\gamma +\mu )\,{\hat{f}}^\beta \big (t,x(\gamma +\mu )\big ) \end{aligned}$$

and then the MMSE estimate of the current value of the basic reproduction rate is given as

24 $$\begin{aligned} {\bar{R}}^0_t={\mathsf{E}}\big [R^{0}_t \,\big |\, (X^{(1)}_u;0\le u\le t) \big ] =\int _0^\infty x {\hat{f}}^R_0(t,x)\, dx. \end{aligned}$$

Note that the expressions of the posterior densities can be used to calculate confidence intervals for all of the above estimates given the path of the observed process ${(X^{(1)}_u; 0\le u\le t)}$, which are directly observed via (8).

## Simulation Study

This simulation study examines the effectiveness of the Bayesian stochastic filtering method described in Section “Bayesian stochastic filtering” in detecting the occurrence of a changepoint in real-time. In a real-time Bayesian setting for changepoint detection, it is essential to decide when to stop and declare that a changepoint has been observed in the data. Let $\tau$ denote the time of stopping and $\theta$ the time of the changepoint in the observed data. Two important measures of effectiveness in this setting are the detection delay, defined as $\max (\tau -\theta ,0)$ and the probability of a false detection (when $\tau <\theta$).

When detecting a change in the drift of a Brownian motion, where drift rates are given and fixed constants known a priori, the optimal stopping time, in terms of minimising detection delay for a bounded probability of false detection, is the first hitting time of the $\Pi$ process (19) to a given threshold $A^*$. This is intuitive, as this means that the first time the probability of a changepoint in the observed data reaches a defined level of confidence, we should stop. In this setting, where the drift rates $b_0$ and $b_1$ are random variables, we will use the same detection approach, i.e. letting $\tau =\inf \{t\ge 0\,|\, \Pi _t \ge A^*\}$. In problems of this type the signal-to-noise ratio (SNR), defined as

$$\text{ SNR }= \left| \frac{b_1-b_0}{\sigma _1}\right|$$

is an important factor which affects the delays and probability of false detections.

In the following simulation study, we consider three scenarios and varying values of the threshold $A^*$ and aim to identify: 1) the detection delays, 2) the proportion of false detections, and 3) the accuracy of the MAP estimate for the changepoint at the time of detection. To do this, we shall simulate paths of the SIR process with the same constants and priors as used with the real data in Section “Application to COVID-19 data”. Letting $\mu =\Lambda =0.01/365$, $\gamma =1/7$, and $\sigma _1 = 0.044$. For each simulation, an exponentially distributed changepoint $\theta$ with parameter $\lambda =0.01$ is generated, and the SIR processes start with a transmission rate $b_0$ which, after time $\theta$, changes to $b_1$. In each simulated path, $b_0$ and $b_1$ are fixed to see how the method performs in various scenarios, which are defined below, however the Bayesian stochastic filtering assumes that the priors for $b_0$ and $b_1$ are uniformly distributed over the interval [0, 0.5] (independent and identically distributed). The simulated data were also sampled at discrete points to mimic the daily data points in the real data considered in Section “Application to COVID-19 data”.

In order to judge how effectively the method works for different signal-to-noise ratios, we consider the following scenarios

25 $$\begin{aligned} \begin{array}{ccc} \text{ Scenario } \text{1: } & (b_0=0.25, b_1=0.206, \sigma _1= 0.044),& \text{ SNR } =1.\\ \text{ Scenario } \text{2: } & (b_0=0.25, b_1=0.162, \sigma _1= 0.044),& \text{ SNR } =2.\\ \text{ Scenario } \text{3: } & (b_0=0.25, b_1=0.118, \sigma _1= 0.044), & \text{ SNR } =3.\\ \end{array} \end{aligned}$$

Exploiting some symmetry in the model and signal-to-noise ratio, these scenarios also provide insight for equivalent sized upward movements in $b_1$. For each scenario, 200 random paths of the SIR process and their associated changepoints are generated. Each path is then studied sequentially and the value of the $\Pi$ process (19) for each new time step is compared to the threshold $A^*$. The first time that the $\Pi$ process is greater than or equal to $A^*$ is the time of detection $\tau$ for that path which is then used to identify the detection delay or if this is a false detection. The MAP estimate for the changepoint at time $\tau$ is then compared to the simulated true value $\theta$.

**Figure 1:**
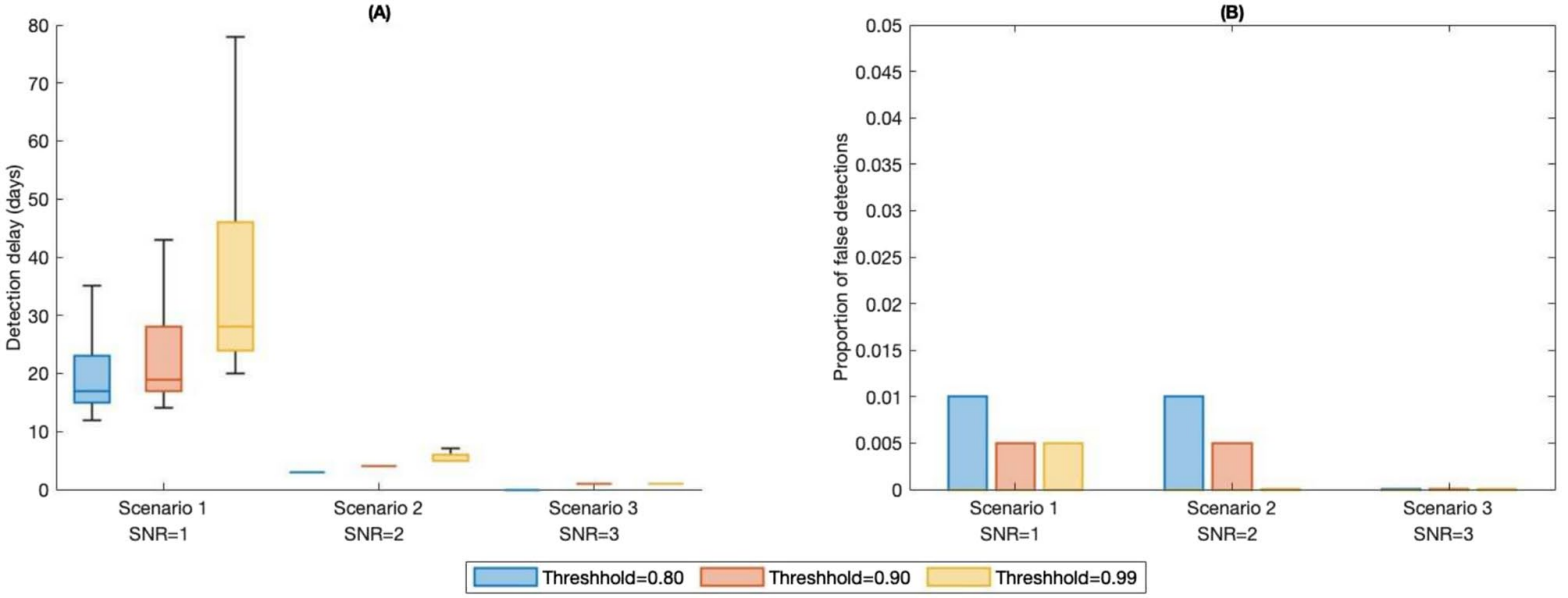
Plots showing the effectiveness of the detection method using Bayesian stochastic filtering described in Section “Bayesian stochastic filtering”. **(A)** Shows the detection delays for 200 simulated paths of the SIR process generated for each scenario described in (25) with varying signal-to-noise ratios. The detection delays are produced for varying thresholds $A^*$, set to 0.8, 0.9, or 0.99. **(B)** Shows the proportion of false detections for the 200 simulated paths of the SIR process used in **(A)**, generated for each scenario described in (25) with varying signal-to-noise ratios. The proportion of false detections is produced for varying thresholds $A^*$, set to 0.8, 0.9, or 0.99.

Figure 1 depicts the resulting detection delays and proportion of false detections for each of the scenarios (25) and varying thresholds $A^*$ of 0.8, 0.9, or 0.99. As the threshold $A^*$ is the level of confidence required to declare that a changepoint has been seen in the data, it is apparent that a higher threshold results in higher detection delays but a lower proportion of false detections. Whereas setting a lower threshold would provide smaller detection delays but at the expense of more false detections. This is what can be seen in Figure 1, where for Scenario 1 with $SNR=1$, the median detection delay is 17 days (IQR 13-23) for $A^*=0.8$; 19 days (IQR 17-28) for $A^*=0.9$; 28 days (IQR 24-46) for $A^*=0.99$. Whereas the proportion of false detections ($\tau <\theta$) was 0.01 for $A^*=0.8$; 0.005 for $A^*=0.9$; 0.005 for $A^*=0.99$.

Finally, for each simulated random SIR process path, the MAP estimate for the changepoint at time $\tau$ is compared to the simulated value of the true changepoint $\theta$. In all 600 simulated random paths of the SIR processes (200 for each of the three scenarios), in every case the MAP estimate of the changepoint at time $\tau$ was exactly the first data point following the changepoint $\theta$. Hence, the MAP estimate of the changepoint at time $\tau$ can be seen to be very accurate in these scenarios. The consistency of this accuracy despite varying signal-to-noise ratios is likely due to the fact that for smaller SNR (e.g. scenario 1) we see longer detection delays which allow for more data to be observed, providing a better estimate of the changepoint, despite the relative change in transmission rate being small. Whereas for high signal-to-noise ratio scenarios (e.g. scenarios 2 & 3) we have shorter detection delays, so fewer data points after the changepoint are observed, but the relative change in transmission rate is larger making the time of the changepoint more apparent.

## Application to COVID-19 data

The following section examines an illustrative example using the described techniques applied to a dataset from the COVID-19 pandemic in the United Kingdom. The data used^30^ includes daily confirmed cases of infection (along with the cumulative number of cases); however, this is obviously an underestimate of infected individuals as it includes only the known cases. Alternative sources of similar data can be found for other countries, for example ^31^. One drawback of this dataset is the lack of data on when people recovered. In a study^32^ on the duration of infectiousness, the median estimate was seven days, during which individuals remain contagious. So, in this illustrative example, similar to other papers^33,34^, we assume that infected individuals recover after 7 days, resulting in a recovery rate of $\gamma =1/7$ with no additional noise or changepoints.

By combining the above data and assumptions with population data^35^, we can observe an estimate of the number of individuals in each compartment, (*S*, *I*, *R*), which we take as representative of the population under consideration. For simplicity, the death rate is set equal to the birth rate, assuming a constant population. In the UK, the crude birth rate is approximately 10 births annually per 1,000 people^36^ so we set $\mu =\Lambda =0.01/365$. We then use (8) to observe the path of $X^{(1)}$, and (10) gives us that $\sigma _1 \approx 0.044$, which being non-zero for this dataset also provides justification for the use of the stochastic model. We also assume that the priors for the unknown transmission rates $b_0,b_1,b_2,\ldots$ are independent and identically distributed with uniform distributions over the interval [0, 0.5]. Similarly, the times between changepoints, $\theta _{i}-\theta _{i-1}$, for $i=1,\ldots ,n$, are independent and identically distributed with an exponential distribution, parameter $\lambda =0.01$.

Using the Bayesian stochastic filtering techniques outlined in Section “Bayesian stochastic filtering”, we can detect changepoints in the observed data. This analysis is performed sequentially, treating the data as if it were appearing in real-time, and the posterior probability of a changepoint being seen in the data (such as (19)) can be compared to a threshold for detection purposes. In the following analysis, we allow for multiple changepoints in the transmission rate within the real data. The detected changepoints can be compared to the dates of public health measures^37^ and the onset of new variants^38^. Users can also reduce the dimensionality of the problem by using the running probability of a changepoint in the data (19) to segment the data into areas containing a single changepoint (cf. method used in ^39^). This approach also means that the user does not need to define the maximum number of changepoints that can be observed in the model.

**Figure 2:**
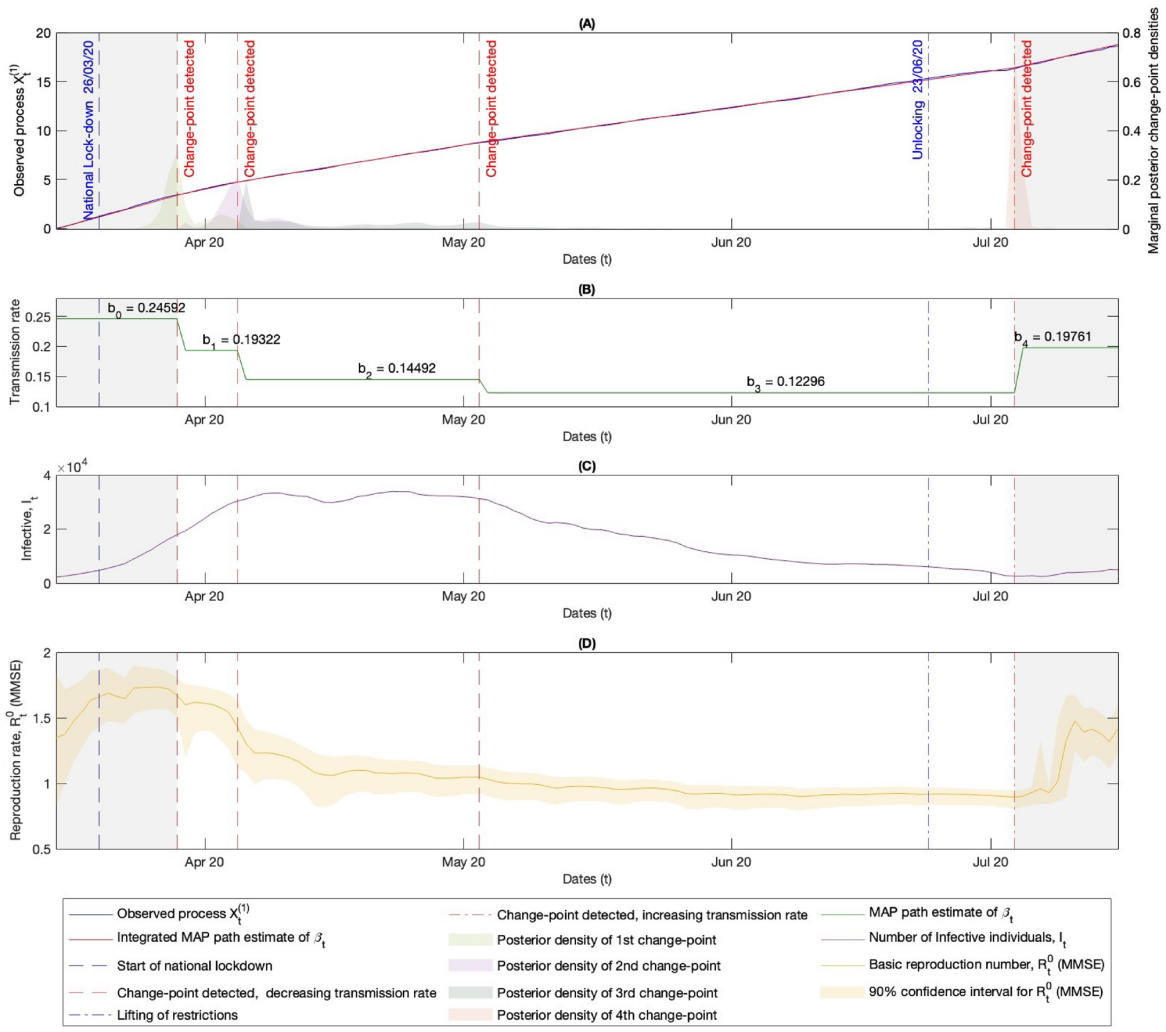
Figures **(A)-(D)** illustrate the times of changes to public health measures relating to the spring 2020 lockdown restrictions in the UK, along with the associated detected MAP estimates for the changepoints in the transmission rate, calculated using the Bayesian stochastic filtering methods discussed in Section “Bayesian stochastic filtering”. **(A)** The left-hand y-axis shows $X^{(1)}$ along with the MAP estimate path for its drift, which is the integrated MAP estimate of the path of $\beta _t$ seen in **(B)**, derived via observation of $X^{(1)}$. The right-hand y-axis shows the posterior densities of the changepoints given the observed data from $X^{(1)}$. **(B)** Shows the MAP estimates for the path of the transmission rate given the observed process $X^{(1)}$ (see (21)), along with the numerical values. **(C)** Shows the path of the number of individuals in the infectious group $I_t$ from the real data. This, along with the number of individuals in the susceptible group $S_t$, is used to derive the path of $X^{(1)}$ seen in **(A)** via (8). **(D)** Shows the real-time MMSE estimate of the current value of the basic reproduction rate (24) and the 90% confidence interval found using the posterior density for $R_t^0$ given in (23).

**Figure 3:**
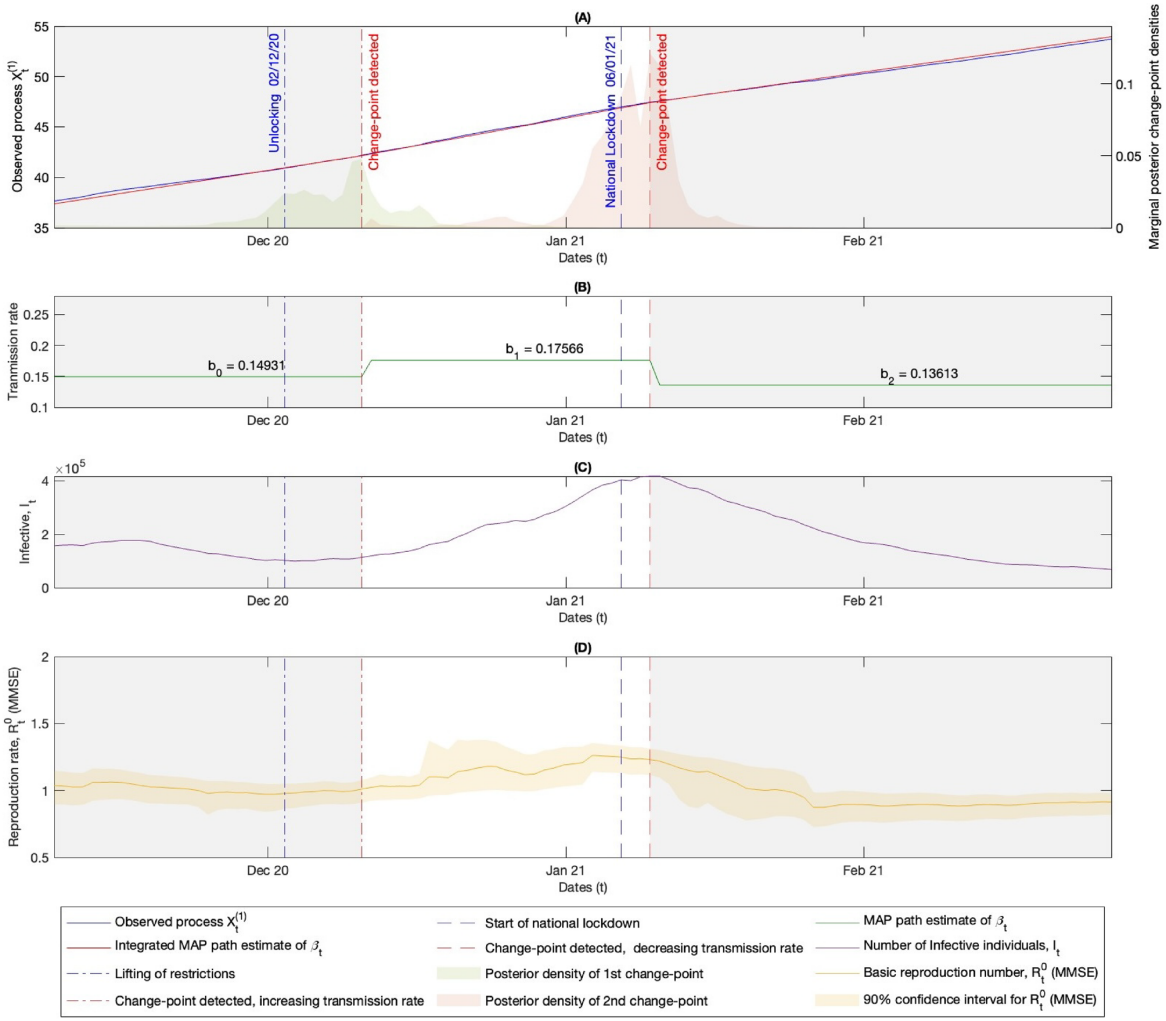
Figures **(A)-(D)** illustrate the times of changes to public health measures relating to the winter 2020/21 lockdown restrictions in the UK, along with the associated detected MAP estimates for the changepoints in the transmission rate, calculated using the Bayesian stochastic filtering methods discussed in Section “Bayesian stochastic filtering”. **(A)** The left-hand y-axis shows $X^{(1)}$ along with the MAP estimate path for its drift, which is the integrated MAP estimate of the path of $\beta _t$ seen in **(B)**, derived via observation of $X^{(1)}$. The right-hand y-axis shows the posterior densities of the changepoints given the observed data from $X^{(1)}$. **(B)** Shows the MAP estimates for the path of the transmission rate given the observed process $X^{(1)}\!$ (see (21)), along with the numerical values. **(C)** Shows the path of the number of individuals in the infectious group $I_t$ from the real data. This, along with the number of individuals in the susceptible group $S_t$, is used to derive the path of $X^{(1)}$ seen in **(A)** via (8). **(D)** Shows the real-time MMSE estimate of the current value of the basic reproduction rate (24) and the 90% confidence interval found using the posterior density for $R_t^0$ given in (23).

**Figure 4:**
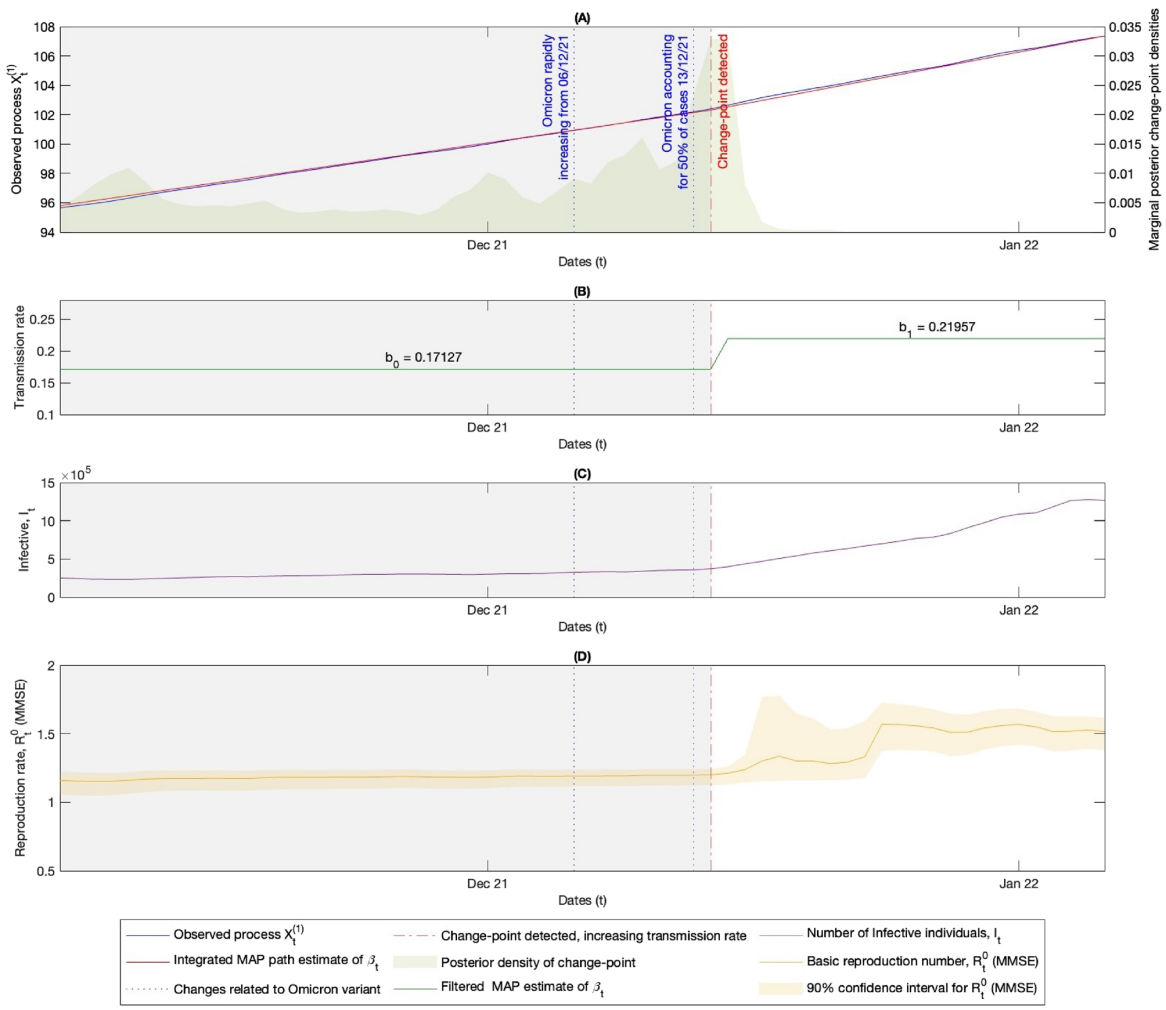
Figures **(A)-(D)** illustrate the times of changes relating to the Omicron variant during the winter of 2021/22 in the UK, along with the associated detected MAP estimate for the changepoint in the transmission rate, calculated using the Bayesian stochastic filtering methods discussed in Section “Bayesian stochastic filtering”. **(A)** The left-hand y-axis shows $X^{(1)}$ along with the MAP estimate path for its drift, which is the integrated MAP estimate of the path of $\beta _t$ seen in **(B)**, derived via observation of $X^{(1)}$. The right-hand y-axis shows the posterior density of the changepoint given the observed data from $X^{(1)}$. **(B)** Shows the MAP estimates for the path of the transmission rate given the observed process $X^{(1)}\!$, see (21), along with the numerical values. **(C)** Shows the path of the number of individuals in the infectious group $I_t$ from the real data. This, along with the number of individuals in the susceptible group $S_t$, is used to derive the path of $X^{(1)}$ seen in **(A)** via (8). **(D)** Shows the real-time MMSE estimate of the current value of the basic reproduction rate (24) and the 90% confidence interval found using the posterior density for $R_t^0$ given in (23).

### Conclusions

Here, we aim to detect changepoints in the transmission rate using data from the United Kingdom, but similar analyses could be conducted for other nations or states. Admittedly, this dataset is quite crude for making population-wide estimates of key quantities. However, despite this limitation, the results in Figures 2-4 demonstrate that the method performs well in detecting changepoints related to public health measures and new variants, serving as a good illustrative example of the outlined methods.

The results in Figures 2 and 3 show that the methods effectively detect changepoints in the transmission rate following changes in public health measures. While the changepoint happening after the public health measure is necessary for a causal relationship which might be expected, here the delay between the time that public health measures are enacted and the time of the detected changepoint in the transmission rate is likely, in part, due to the lack of an incubation period in the SIR model considered. The incubation period is the latency between getting infected, showing signs of infection, and becoming infectious to others, which for COVID-19 was estimated to be around six days^40^ on average. This latency implies that the changepoint would not be observed in the data for approximately six days if a public health measure caused a change in the transmission rate, as individuals typically test for the disease only when showing signs of infection. This would account for some of the delay in detecting the changepoints. Figure 4 shows how the method detects an increase in the transmission rate on the 14th of December 2021, which closely relates to the emergence of the Omicron wave. The Omicron variant rapidly increased from 6th of December, accounting approximately for 50% of cases of COVID-19 by 13th of December 2021^38^. This further shows how this method could be used to provide useful information about new variants.

These ideas could be easily extended to allow for the rates to be more complicated functions of time to allow for events such as detecting behavioural fatigue causing people to begin to ignore public health measures. In such case, the changepoint may be the start of a more gradual transition from one transmission rate to another (see ^23^ for comparison of existing methods). This could also include better modeling of the natural seasonality in the transmission and recovery rates, allowing for transmission rates to naturally vary over the course of the week around the current baseline level, and be used to answer related questions.

The true strength of this method is in its ability in real-time detection and identification of unknown rates in the model. The Bayesian setting allows users to quantify and update their beliefs about the underlying distributions of unobservable quantities in the model. The analysis for detecting and quantifying the probability of changepoints (using (19) or similar for multiple changepoints) and current MMSE estimates could be performed in real-time to inform public health decisions, such as measuring the effectiveness of national restrictions and providing useful information about new disease variants.

## Data Availability

The datasets analysed during the current study are available in the repository ^30^, which can be found at https://github.com/CSSEGISandData/COVID-19.
